# Family involvement along the care continuum for small and sick newborns – attitudes and skills of healthcare providers in Ghana

**DOI:** 10.1186/s41043-025-01001-2

**Published:** 2025-08-04

**Authors:** Christina Schuler, Faith Agbozo, Emmanuel Bansah, Richard Owusu, George Edward Ntow, Barbara Preusse-Bleuler, Riccardo E. Pfister

**Affiliations:** 1 Institute of Global Health, Faculty of Medicine, University Geneva, Geneva, Switzerland; 2 Institute of Nursing, School of Health Sciences, ZHAW Zurich University of Applied Sciences, Winterthur, Switzerland; 3 Department of Family and Community Health, Fred N. Binka School of Public Health, University of Health and Allied Sciences, Ho, Ghana; 4 Volta Regional Hospital, Hohoe, Ghana; 5 Dodowa Health Research Centre, Dodowa, Ghana; 6 Neonatal and Pediatric Intensive Care Unit, University Hospitals of Geneva and Geneva University, Geneva, Switzerland

**Keywords:** Family centered care, Patient participation, Newborn, Pregnancy, Family Nursing, Ghana, Health Personnel, Practice skills

## Abstract

**Background:**

The high social and emotional burden on families with small or sick newborns necessitates family-focused care. Positive attitudes towards family involvement and skilled healthcare providers are crucial for improving the health and well-being of small/sick newborns and their families. While family systems care (FSC) has been explored and implemented in high-income countries, it is rarely reported in low- and middle-income countries. This study aims to assess knowledge, attitudes and skills of healthcare providers on FSC and the family care practices, interactions, and reciprocity in the provider-family relationship throughout the care continuum of sick/small infants and their families.

**Methods:**

A cross-sectional survey involved healthcare providers offering maternal and newborn care at secondary and primary-level facilities. Participants were sampled from one secondary-level hospital and 13 primary health facilities in the Hohoe Municipality of Ghana. The survey instruments included the standardized and validated instruments ‘Family Importance in Care - Nurses’ attitudes’ (FINC-NA) and ‘Family Nursing Practice Scale’ (FNPS), which measure healthcare providers’ attitudes and skills in working with families, alongside four open-ended questions. Quantitative data were analyzed using descriptive statistics, while answers to open-text components were categorized thematically.

**Results:**

Overall, 143 healthcare providers, comprising nurses, midwives and physicians, participated in the study. The median score of FINC-NA was 93 (83–99), while the FNPS was 30 (24–35). Most healthcare providers had a positive attitude towards family involvement in care, whereas practice skills were moderate. From the open-ended questions, care providers’ perceptions and actions in relation to family care practices were communication difficulties, intra-family challenges and confidentiality concerns. They also reported reduced workload, stronger healthcare provider-family relationship and improved quality of care through increased family collaboration and support.

**Conclusions:**

Overall, healthcare providers valued family involvement in the care but had only moderate skills in working with them. Advocacy for the inclusion of family systems care in healthcare education, in-service training and policy development in Ghana is warranted. This will support healthcare providers to deliver high-quality care to families with small or sick newborns from pregnancy to post-discharge.

**Supplementary Information:**

The online version contains supplementary material available at 10.1186/s41043-025-01001-2.

## Background

A baby born small (< 2500 g) or sick can bring significant social, financial, and emotional burdens to the family and society (Ashorn et al. 2023; WHO 2019). This burden can have additional adverse effects on the newborns’ developmental, social and cognitive growth (Moxon et al. 2015; WHO 2019). In 2020, one in every ten newborns was born premature (< 37 weeks gestational week) and one in five was born small for gestational age with the greatest burden recorded in sub-Saharan Africa (SSA) and Southern Asia (Lawn et al. 2023). Globally, every year, over 30 million newborns need inpatient hospitalization, and over 1.3 million survive with major disabilities (WHO 2019, 2024a). However, the full magnitude of neonatal morbidity and disability in low- and middle-income countries (LMICs) remains largely underreported and unknown (Ashorn et al. 2023; Rosa-Mangeret et al. 2022).

Nurturing care provides a platform for responsive interactions that are emotionally supportive and developmentally stimulating. This lays the foundations for critical elements of our next generation’s health, well-being and productivity with long-lasting effects (Black et al. 2021; WHO et al. 2018). Healthcare providers play a vital role in providing tailored nurturing during pregnancy and birth preparation, along with ongoing support for the care of vulnerable newborns (Black et al. 2021; WHO and UNICEF 2021). This nurturing care fosters lifelong bonding between parents and their newborns, facilitating physical and neurological development (Maria and Agrawal 2021). Therefore, empowering families to offer nurturing and affectionate care and appropriate sensory interaction to their newborns should be a primary goal of healthcare providers (Maria et al. 2021).

In recent years, the role of families in healthcare has received more attention. In 2018, the WHO conceptualized and adopted the nurturing care framework placing families at the center of attention to guide on how to engage them in the care of their newborn (WHO et al. 2018). In 2020, family-centered care was adopted as one of the eight World Health Organization’s “standards for the care of small and sick newborns in health facilities” (WHO 2020). These standards place the families at the core of every care delivery and are particularly relevant for newborns with special care needs, as their rights are often overlooked, and they frequently lack respectful care (Maria and Agrawal 2021; Rosa-Mangeret et al. 2022).

Family involvement is strongly influenced by healthcare providers’ attitudes towards the role and importance of families in care (Barreto et al. 2022). An attitude can be defined as ‘an evaluation of a psychological object, represented along dimensions such as good versus bad or likable versus unlikable’ (Ajzen 2001). Positive attitudes of healthcare providers towards families have been shown to improve their engagement across the care continuum (Benzein et al. 2008b; Hsiao and Tsai 2015). The attitude of healthcare providers towards the role and importance of families in the care of their family members is essential and a starting point for implementing a family systems approach to care (Hagedoorn et al. 2021). The relationship between healthcare providers and families should be reciprocal, evolving through interactions among the healthcare providers, patient and family. The relationship is characterized by the health providers’ thoughtful care planning and efforts to encourage family involvement and mutual exchange (Simpson et al. 2006).

However, power imbalances and disrespectful attitudes continue to hinder the development of a positive relationship between healthcare providers and families, posing significant barriers to family inclusion (Mirlashari et al. 2020). Studies from Africa reported disrespectful care and poor rapport between patients and healthcare providers across antenatal (Lythgoe et al. 2021), delivery (Kassa et al. 2020) and postnatal phases (Abukari and Schmollgruber 2024).

The concept of family-centered care emphasizes the involvement of families in the care of their newborns (Ndwiga et al. 2022). Family systems care (FSC) extends this notion (Bell 2013) and prioritizes dignity, respect and shared decision-making. It positions families and healthcare providers as collaborative partners (Shajani and Snell 2023). FSC facilitates tailored assessments and ongoing support for psychosocial needs as it considers the human being as a physical-mental being in the eco-social living environments thereby contributing significantly to health promotion, prevention, and support during illness (Shajani and Snell 2023; WHO 2019). It enables healthcare providers to consider the family in interaction and reciprocity between multiple systems, within the family itself, society and the healthcare system (Bell 2009). FSC enables families of small/sick newborns to provide sustained neurodevelopmental and nurturing care from the onset of pregnancy throughout birth and the postnatal period (Ndwiga et al. 2022; WHO 2018; Zahara Shajani and Snell 2023). FSC enhances family functioning and quality of life in hospital and home care settings (Moules et al. 2016; WHO 2018) by alleviating stress, anxiety and improving symptom and everyday life management (O’Brien et al. 2018; Shajani and Snell 2023) examples being higher breastfeeding rates and neonatal weight gain (Mushtaq and Kazi 2019; O’Brien et al. 2018). Additionally, it enhances work satisfaction and resource allocation in healthcare institutions (Maria et al. 2021; Verma et al. 2017). In a nutshell, a good family-healthcare provider relationship is mutually beneficial (Barreto et al. 2022; Simpson and Tarrant 2006).

To date, most research on this topic has emanated from high-income countries (Barreto et al. 2022). Particularly Canada (Hoplock et al. 2019), USA (Misto 2018), Sweden (Gusdal et al. 2017), and Portugal (Fernandes et al. 2018), with a few from LMICs, such as Brazil (Boyamian et al. 2021) and Uganda (Imanipour and Kiwanuka 2020). Globally, studies on family-centered care focus on neonatal units, often excluding antenatal, intrapartum and community care (Abukari and Schmollgruber 2023; Tekelab et al. 2019). Broader involvement will bridge the gap between facility- and home-based newborn care (Maria and Agrawal 2021).

In Africa, the family holds significant importance (Adjei et al. 2024; Imanipour and Kiwanuka 2020). Despite the family importance in Ghana, family integration in healthcare is hindered by incoherent policies and training opportunities (Ohene et al. 2019; Schuler et al. 2023a). In SSA, this is particularly manifest for antenatal and intrapartum care (Chironda et al. 2022; Landry et al. 2023). There is a lack of evidence on how healthcare providers can or should enable family involvement across the care continuum of small/sick newborns (Anyanwu et al. 2024; Schuler et al. 2023a). Although FSC has proven effective to enhance neonatal care in high-income contexts, such evidence is scarce in the low-income African settings, including Ghana. Contextual assessments of current practice skills and attitudes of healthcare providers, particularly along maternal and newborn care, are exceedingly rare or non-existent.

### Research objectives

The goal of this study was to generate baseline data throughout the care continuum of sick/small infants and their families to implement a family systems care program. We determined the knowledge, attitudes and skills of healthcare providers on family care practices, interactions and reciprocity in the healthcare provider-family relationship, including current challenges.

## Methods

### Design

This cross-sectional study was based on an online survey. The survey was used, adhering to the Strengthening the Reporting of Observational Studies in Epidemiology (STROBE) guidelines for articles reporting cross-sectional studies (STROBE-Initiative 2024).

### Setting

This study took place in the Hohoe Municipality, eastern Volta Region, Ghana, a region comprised mainly of small communities with unreliable road and telecommunications access.

Most engage in crop and livestock farming and petty trading, and the literacy rate is 49% (Ghana Health Service 2023; Ministry of Food and Agriculture 2023).

The Municipality has a secondary-level referral hospital (regional level II hospital) offering care to over 200,000 inhabitants comprising cesarean sections, neonatal intensive care and postpartum services. Staffing includes general nurses, a few specialist nurses, midwives, house officers, general physicians, as well as one pediatrician and one obstetrician.

A Public Health and Nutrition Unit (PHNU), which offers child welfare services, such as growth monitoring, vaccination, and nutrition counseling, sometimes acts as a liaison between the NICU and primary care facilities (Schuler et al. 2023a).

At the primary level, health care is administered through eight health centers and five CHPS (Community-Based Health Planning and Services) zones staffed by community health nurses and volunteers providing basic maternal, neonatal and child health care, breastfeeding support, growth monitoring, vaccination, management of minor ailments and referrals services. Depending on the size, midwives and physician assistants occasionally complement the team. A newly established telecommunication unit of the Volta Regional Hospital manages referrals between the various levels of care.

### Participants

Our survey comprised healthcare providers working with pregnant or laboring women and/or small/sick newborns and their families. We included nurses, midwives, physician assistants and physicians. The study participants were either working at the antenatal care clinic, labor ward, recovery ward, neonatal intensive care unit, postnatal ward, the hospitals’ PHNU or at one of the 13 primary care facilities. We excluded students and community volunteers undergoing basic vocational training as we aimed to capture the experience of professionals with completed professional training.

### Sample size

The population census approach was used to determine the sample size (Israeli 1992). At the time of the study there were 208 healthcare providers working in the various units of the hospital and primary care structures. For this limited number of providers, we decided to invite all healthcare providers involved in maternal and newborn care.

### Data sources/measurement

The data collection was between June 2023 and August 2023, using four different validated scales. Here we report findings from the following scales: Family Importance in Nursing Care-Nursing Care (FINC-NA) (Benzein, Johansson, Arestedt, Berg, et al., 2008) and the Family Nursing Practice Scale (FNPS) (Simpson and Tarrant 2006). The other two scales, which investigated implementation outcomes and context analysis, are reported separately. Unlike the FNPS, which is freely available online, permission to use the FINC-NA was obtained from authors of the original tool.

The FINC-NA, which has 26 items in four subsections (Table 1), was used in its original English version to assess healthcare providers’ attitude towards the importance of family involvement in care. Responses are graded on a five-option Likert scale from strongly disagree (score 1) to strongly agree (score 5). The total score ranges from 26 (minimum) to 130 (maximum), with higher scores reflecting more positive attitudes towards family importance in care (Benzein, Johansson, Arestedt, Berg, et al., 2008). The tool has good psychometric properties with an internal consistency of 0.88 Cronbach’s alpha (Benzein, Johansson, Arestedt, Berg, et al., 2008).

The FNPS tool consists of 10 items, which are also ranked on a 5-point Likert scale ranging from 10 (minimum) to 50 (maximum), with a lower score indicating higher skills (Table 1). The FNPS tool has an overall reliability of 0.86 Cronbach’s alpha (Simpson and Tarrant 2006).

**Table 1:** Definition and interpretation of the scales used for data collection

Subscales	Definition	Minimum-Maximum Score
**Family Importance in Nursing Care – Nurses Attitudes (FINC-NA)**		
Family as a Resource in Nursing Care (FAM-RNC)	Positive attitude towards families and the value of their presence in care	10–50
Family as a Resource in Nursing Care (FAM-CP)	Importance of acknowledging the patient’s family members and having a dialogue with them	8–40
Family as a Burden (FAM-B)	Negative statements about the family	4–20
Family as is Own Resource (FAM-OR)	Acknowledging families as having their own resources for coping	4–20
***FINC-NA Total score***		**26–130**
**Family Nursing Practice Scale (FNPS)**		
Practice appraisal (PA)	Represents the healthcare providers critical appraisal of their own family care practice in terms of confidence, satisfaction, knowledge, skill, and comfort	5–25
Nurse family relationship (NFR)	Healthcare providers assessment of their own family – healthcare provider relationship	5–25
***FNPS Total score***		**10–50**

*FINC-NA: Family Importance in Nursing Care – Nurses Attitudes

*FNPS: Family Nursing Practice Scale

The scale also includes three open-ended questions on perception of work with families, which was complemented by a fourth question as presented below. Pre-testing of the instruments generated feedback on the sociodemographic variables and length of the questionnaire. Like Imanipour and Kiwanuka (2020), who used the scales in a similar context in Uganda, no concerns were raised about the clarity or appropriateness.

### Open-text components

The following three FNPS open-text components were completed with a fourth one:

1. *What problems or drawbacks are there in your care practice when involving the family in assessment and care planning;*
2. *What advantages, if any, are there for your nursing/care practice when involving the family in assessment and care planning*;
3. *What have you done in the past week to involve families in your current care practice;*
4. *Is there anything else you think is important we have not asked?*

### Procedure and data management

The questionnaires were self-administered using Kobo Collect (2023), an open-source software for online surveys. An online link to the questionnaire was shared with eligible participants through the supervisors of the various care units. The generated quantitative data was downloaded into Microsoft Excel, cleaned, and exported into Stata version 17 for analysis.

### Analysis

We reported demographics using frequencies and proportions. Descriptive statistics included median and interquartile range as the data was skewed. Overall and sub-scores of the FINC-NA and FNPS are reported with absolute numbers for each unit of care and combined for all units across the care continuum. For each instrument, Cronbach’s alpha coefficient of reliability was calculated to test internal consistency.

Open-text responses were categorized thematically using Excel. Divergent ideas were discussed among the researchers until a common understanding was reached.

### Ethical considerations

The declaration of Helsinki guided the study approach (World Medical Association 2024). Ethical clearance was granted by the Ghana Health Service Ethical Review Committee (GHS-ERC 027/03/23). Permission and administrative approval were obtained from the Volta Regional Hospital Management and the Hohoe Municipal Health Directorate. Study information was provided via the online link and participants consented online prior to participation. Analysis was anonymous.

## Results

### Participant characteristics

A total of 143 healthcare providers participated in the survey (response rate of 68.8%), mostly females (115; 80.4%), and the mean age was 33 years (SD ± 5.0). Diploma was the highest educational level of 69 (48.6%). The mean work experience in the units was 5.0 years (SD ± 3.7) and 76.9% had experienced serious illness of a family member. Also, 62.2% stated the availability of a family systems policy in the care of families at their workplace, but there were varying opinions within wards about whether this policy was actually implemented (Table 2).

**Table 2:** Health providers’ demographics along the eight wards of the continuum of care [n (%)]

		Overall	ANC	Labor ward	Recovery ward	NICU	Postnatal ward	PHNU	Health centers	CHPS
		*N* = 143 (100)	*N* = 10 (7.0)	*N* = 17 (11.9)	*N* = 2 (1.3)	*N* = 17 (11.9)	*N* = 9 (6.3)	*N* = 8 (5.6)	*N* = 54 (37.8)	*N* = 26 (18.2)
**Age (Years)**	(Mean/SD)	33 (± 5.0)	34 (± 6.0)	34 (± 6.8)	31 (± 1.4)	32 (± 2.7)	31 (± 4.1)	34 (± 7.1)	32 (± 4.9)	33 (± 5.0)
	20–29	36 (25.2)	2 (20.0)	1 (5.9)	0 (0.0)	4 (23.5)	3 (33.3)	1 (12.5)	20 (37.0)	5 (19.2)
	30–39	96 (67.1)	7 (70.0)	15 (88.2)	2 (100)	13 (76.5)	5 (55.7)	6 (75.0)	29 (53.7)	19 (73.1)
	40+	11 (7.7)	1 (10.0)	1 (5.9)	0 (0.0)	0 (0.0)	1 (11.1)	1 (12.5)	5 (9.3)	2 (7.7)
**Gender**	Female	115 (80.4)	10 (100)	16 (94.1)	2 (100)	11 (64.7)	8 (88.9)	7 (87.5)	42 (77.8)	19 (73.1)
**Professions**	CHN	38 (26.6)	0 (0.0)	0 (0.0)	0 (0.0)	0 (0.0)	0 (0.0)	4 (50.0)	17 (31.5)	17 (65.4)
	General nurse	30 (21.0)	0 (0.0)	0 (0.0)	2 (100.0)	11 (64.7)	1 (11.1)	0 (0.0)	14 (25.9)	2 (7.7)
	Enrolled nurse	16 (11.2)	3 (30.0)	0 (0.0)	0 (0.0)	2 (11.8)	0 (0.0)	3 (37.5)	5 (9.3)	3 (11.5)
	Pediatric Nurse	3 (2.1)	0 (0.0)	0 (0.0)	0 (0.0)	3 (17.7)	0 (0.0)	0 (0.0)	0 (0.0)	0 (0.0)
	Midwife	51 (35.7)	7 (70.0)	16 (94.1)	0 (0.0)	0 (0.0)	8 (88.9)	1 (12.5)	16 (29.6)	3 (11.5)
	Medical staff*	5 (3.5)	0 (0.0)	1 (5.9)	0 (0.0)	1 (5.9)	0 (0.0)	0 (0.0)	2 (3.7)	1 (3.8)
**Education**	Certificate	47 (32.4)	0 (0.0)	0 (0.0)	0 (0.0)	3 (17.7)	1 (11.1)	3 (37.5)	20 (37.7)	19 (73.1)
	Diploma	69 (48.6)	6 (60.0)	9 (52.9)	1 (50.0)	10 (58.8)	6 (66.7)	3 (37.5)	28 (52.8)	6 (23.1)
	Bachelor	21 (14.8)	1 (10.0)	7 (41.2)	1 (50.0)	2 (11.8)	2 (22.2)	2 (25.0)	5 (9.4)	1 (3.8)
	Masters	3 (2.1)	2 (20.0)	0 (0.0)	0 (0.0)	1 (5.9)	0 (0.0)	0 (0.0)	0 (0.0)	0 (0.0)
	Other	3 (2.1)	1 (10.0)	1 (5.9)	0 (0.0)	1 (5.9)	0 (0.0)	0 (0.0)	0 (0.0)	0 (0.0)
**Work experience in the unit**	(Mean/SD)	5 (± 3.7)	6 (± 4.6)	8 (± 4.3)	2 (± 0.7)	4 (± 2)	5 (± 2.8)	5 (± 3.2)	4 (± 3.5)	5 (± 4.0)
**(years)**	0–3	101 (70.6)	10 (100.0)	8 (47.1)	2 (100.0)	13 (76.5)	6 (66.7)	3 (37.5)	40 (74.1)	19 (73.1)
	4–9	37 (25.9)	0 (0.0)	8 (47.1)	0 (0.0)	4 (23.5)	3 (33.3)	5 (62.5)	11 (20.4)	6 (23.1)
	10+	5 (3.5)	0 (0.0)	1 (5.9)	0 (0.0)	0 (0.0)	0 (0.0)	0 (0.0)	3 (5.5)	1 (3.8)
**Previous history of family member with serious illness**	Yes No	58 (40.6) 85 (59.4)	5 (50.0) 5 (50.0)	9 (52.9) 8 (47.1)	2 (100) 0 (0.0)	7 (41.2) 10 (58.8)	5 (55.6) 4 (44.4)	4 (50.0) 4 (50.0)	18 (33.3) 36 (66.7)	8 (30.8) 18 (69.2)
**Availability of general approach to the care of families at place work**	Yes No	89 (62.2) 54 (37.8)	5 (50.0) 5 (50.0)	12 (70.6) 5 (29.4)	1 (50.0) 1 (50.0)	10 (58.8) 7 (41.2)	6 (66.7) 3 (33.3)	6 (75.0) 2 (25.0)	35 (64.8) 19 (35.2)	14 (53.8) 12 (46.2)

ANC = Antenatal care, CHN = Community Health Nurse, NICU = Neonatal Intensive Care Unit, PHNU = Public Health and Nutrition Unit, CHPS = Community-based health planning and services

*Medical staff included 2 general physicians, 2 physician assistants, 1 medical assistant

### Healthcare providers’ attitudes towards the importance of families in care

The overall median score with the corresponding IQR on the FINC-NA scale (max 130) was 93 (83–99) (Table 3). Across the different units, the median varied. The lowest score was at the CHPS (88 IQR:77–116) and the highest at the Public Health and Nutrition Unit (PHNU) (100 IQR:85.5-104.5). The IQR for the healthcare providers’ attitudes towards family as a resource in nursing care (FAM-RNC; max 50) was 39 (34–41). Staff of the NICU and the health centers had the most positive perception of families being a resource in care. Compared to other wards, families were least likely to be a conversational partner (FAM-CP) at the recovery ward (20.5 IQR:29–32). The median score for the subscale “Family as a burden” was 9 (8–11), which indicates that healthcare providers perceived family as a moderate burden in care. The PHNU and the postnatal ward had the highest scores, indicating that they perceived families less of a burden than the other wards. The subscale “family as own resource (FAM-OR)” had a total median score of 16 (14–16), meaning that healthcare providers believed that families are resource to themselves.

**Table 3:** Summary score for family importance in nursing care- nursing attitude FINC-NA subscale and total score

	Overall	ANC	Labor Ward	Recovery Ward	NICU	Postnatal Ward	PHNU	Health Centers	CHPS
		**Median (IQR)**							
**Family as a Resource in Nursing Care (Fam-RNC)** *(Min.10 - Max. 50)*	39 (34–41)	38.5 (30–40)	37 (34–40)	37 (34–40)	40 (40–42)	38 (34–39)	39.5 (34–42)	40 (36–44)	37.5 (32–40)
**Family as a Conversational Partner (Fam-CP) (** *Min.8 - Max. 40)*	30 (26–32)	29 (24–32)	29 (26–31)	20.5 (29–32)	32 (29–32)	28 (28–30)	32 (27–32.5)	30.5 (26–32)	29 (22–32)
**Family as a Burden (Fam-B)** *(Min. 4 – Max. 20)*	9 (8–11)	10 (9–10)	10 (8–11)	8.5 (7–10)	9 (7–11)	11 (9–11)	12.5 (11–14)	8.5 (7–10)	10 (8–11)
**Family as Own Resource (Fam-OR)** (*Min. 4 – Max. 20)*	16 (14–16)	15.5 (14–16)	15 (13–16)	15 (14–16)	16 (14–16)	14 (13–15)	15 (13–16.5)	16 (14–17)	14.5 (12–16)
**Total score FINC-NA** *(Min. 26 – Max. 130)*	93 (83–99)	92 (81–101)	90 (82–95)	91 (84–98)	95 (92–99)	93 (83–95)	100 (85.5–104.5)	94 (85–102)	88 (77–116)

a Families’ Importance in Nursing Care – Nurses’ Attitudes, high score = positive attitudes

Table 4 shows the median scores and corresponding IQR for the FINC-NA, providing information on healthcare providers’ attitudes towards the family’s importance in care. Overall, all four subscales recorded a high score.

**Table 4:** Median score for family importance in nursing care- nursing attitude (FINC-NA)

	ANC	Labor Ward	Recovery Ward	NICU	Postnatal Ward	PHNU	Health Centers	CHPS	
	Median (IQR)								
**Family as a Resource in Nursing Care (Fam-RNC)**									
A good relationship with family members gives me job satisfaction	4 (4–4)	4 (4–5)	3.5 (3–4)	4 (4–5)	4 (4–4)	4 (3.5–4)	4 (4–5)	4 (3–4)	
Family members should be invited to actively take part in the patient’s care	4 (4–4)	4 (4–4)	4.5 (4–5)	4 (4–5)	4 (4–4)	4 (3–4.5)	4 (4–5)	4 (4–4)	
The presence of family members is important to me as a professional	4 (4–4)	4 (4–5)	4 (4–4)	4 (4–5)	4 (4–4)	4 (3.5–4)	4 (4–5)	4 (3–4)	
The presence of family members gives me a feeling of security	3 (2–4)	3 (3–4)	3 (2–4)	4 (3–4)	4 (2–4)	4 (3.5–5)	4 (3–4)	4 (3–4)	
The presence of family members eases my workload	3.5 (3–4)	3 (2–4)	3 (2–4)	4 (4–5)	4 (3–4)	4 (3–4)	4 (3–4)	4 (3–4)	
Family members should be invited to actively take part in planning patient care	4 (4–4)	4 (3–4)	4 (4–4)	4 (4–5)	4 (2–4)	4 (3.5–5)	4 (3–5)	4 (3–4)	
The presence of family members is important for the family members themselves	4 (4–4)	4 (4–4)	4 (4–4)	4 (4–5)	4 (4–4)	4 (3.5–4)	4 (3–4)	4 (3–4)	
Getting involved with families gives me a feeling of being useful	4 (3–4)	4 (3–4)	3.5 (3–4)	4 (4–4)	4 (2–4)	4 (3.5–4)	4 (4–5)	4 (4–4)	
I gain a lot of worthwhile knowledge from families which I can use in my work	4 (4–4)	3 (3–4)	3.5 (3–4)	4 (3–4)	4 (3–4)	3.5 (2.5–4)	4 (4–4)	4 (3–4)	
It is important to spend time with families	4 (3–4)	4 (3–4)	4 (4–4)	4 (4–4)	4 (4–4)	4 (3.5–4.5)	4 (4–4)	4 (3–4)	
**Family as a Conversational Partner (Fam-CP)**								**)**	
It is important to find out what family members a patient has	4 (4–4)	4 (4–4)	4 (4–4)	4 (4–5)	4 (4–4)	4 (3–5)	4 (3–4)	3 (2–4)	
I ask family members to take part in discussions from the very first contact	3.5 (2–4)	4 (3–4)	4 (4–4)	4 (4–4)	4 (4–4)	4 (3.5–4.5)	4 (3–4)	4 (2–4)	
Discussion with family members during first care contact saves time	4 (4–4)	4 (3–4)	4 (4–4)	4 (4–4)	4 (3–4)	4 (3.5–4.5)	4 (3–4)	3.5 (3–4)	
I always find out what family members a patient has	3.5 (3–4)	4 (3–4)	3.5 (3–4)	4 (3–4)	3 (2–4)	3.5 (3–4)	4 (2–4)	4 (2–4)	
I invite family members to have a conversation at the end of the care period	4 (3–4)	4 (3–4)	4 (4–4)	4 (4–4)	4 (3–4)	4 (3–4.5)	4 (4–4)	4 (3–4)	
I invite family members to actively take part in the patient’s care	3.5 (2–4)	4 (3–4)	4 (4–4)	4 (4–4)	4 (4–4)	4 (3.5–4)	4 (3–4)	4 (2–4)	
I invite family members to speak about changes in the patient’s condition	4 (4–4)	4 (4–4)	3 (2–4)	4 (4–4)	4 (2–4)	4 (3–4)	4 (4–5)	4 (3–4)	
I invite family members to speak when planning care	4 (3–4)	3 (3–4)	4 (4–4)	4 (4–4)	4 (3–4)	4 (3.5–4)	4 (4–4)	4 (3–4)	
**Family as a Burden (Fam-B)**:									
The presence of family members holds me back in my work	4 (3–4)	4 (3–5)	4 (4–4)	4 (3–5)	4 (4–4)	3 (2–3)	4 (4–4)	4 (3–5)	
I do not have time to take care of families	4 (3–4)	4 (4–4)	4 (4–4)	4 (4–5)	4 (3–4)	4 (3–4.5)	4 (4–5)	4 (3–5)	
The presence of family members makes me feel that they are checking up on me	3 (2–4)	3 (3–4)	3 (2–4)	3 (2–4)	2 (2–4)	2.5 (2–3)	3 (2–4)	2 (2–3)	
The presence of family members makes me feel stressed	4 (3–4)	3 (4–5)	4.5 (4–5)	4 (4–4)	4 (3–4)	3 (2.5–3.5)	4 (4–4)	4 (3–5)	
**Family as Own Resource (Fam-OR)**									
I ask families how I can support them	4 (3–4)	4 (4–4)	4 (4–4)	4 (3–4)	3 (2–4)	4 (3–4)	4 (3–4)	4 (2–4)	
I encourage families to use their own resources	4 (3–4)	4 (3–4)	3 (2–4)	4 (4–4)	4 (4–4)	4 (3–4)	4 (4–5)	4 (3–4)	
I consider family members as cooperating partners	4 (4–4)	4 (3–4)	4 (4–4)	4 (4–4)	4 (3–4)	4 (3.5–4)	4 (4–5)	4 (4–4)	
I see myself as a resource for families so that they can cope	4 (3–4)	4 (3–4)	4 (4–4)	4 (4–5)	4 (3–4)	4 (3.5–4)	4 (4–4)	4 (4–4)	

Concerning the family as a resource in nursing care (Fam-RNC), most healthcare providers had a median score of 4 (out of 5), indicating that they perceived families as a resource in the care provision. This level of attitude was consistent across all units of care.

In the subscale on conversational partner (FAM-CP), although few of the healthcare providers remained neutral, most had a median score of 4, indicating they viewed family as a conversational partner.

For the subscale on family as a burden (FAM-B), some healthcare providers remained neutral to the negative statements towards families, such as *“The presence of family members make me feel that they are checking up on me”*, while others perceived families as a burden. A high score was observed for the statement: “*I do not have time to take care of families”.*

The family as an own resource (FAM-OR) subscale showed mostly positive responses with a median score of 4, indicating that healthcare workers considered families to have their own resources for coping. Most healthcare providers also considered families as cooperating partners, as supported by a median score of 4 for the question *“I consider family members as cooperating partners”.*

### Healthcare providers’ appraisal of practice skills and reciprocity

Table 5 details healthcare providers’ practice skills and the healthcare provider-family relationship. Lower scores indicate more effective practice or a stronger relationship. The overall score on the FNPS scale was 30 out of a maximum of 50. The highest practice skills were observed in the PHNU (21.5) and lowest in the health center (28). The lowest practice skills were observed for the ANC (34) and labor ward (34), followed by the CHPS (33) and the NICU (32). In the subscale practice appraisal (PA), healthcare providers at the PHNU (11) and recovery ward (12.5) showed the highest critical appraisal of their own family care practice. The lowest skills were reported from the labor ward (17) and the CHPS compounds (17). The PHNU had the highest interaction and reciprocity in healthcare provider-family relationship (10.5), followed by the health centers (13.5). The lowest relationship competencies were reported from the labor ward (16) and the ANC (15.5), both showing moderate healthcare provider-family relationship.

**Table 5:** Median scores for healthcare providers’ skills in working with families (FNPS) (*n* = 143)

Skills/reciprocity in working with families	Overall	ANC	Labor ward	Recovery ward	NICU	Postnatal ward	PHNU	Health center	CHPS
	**Median (IQR)**								
Practice appraisal (PA) *(Min 5 – Max 25)*	15 (12–18)	15.5 (11–20)	17 (16–19)	12.5 (8–17)	15 (14–17)	14 (13–15)	11 (9.5–16.5)	15 (11–17)	17 (13–19)
Health provider -family relationship (NFR) (*Min.5 – Max.25)*	15 (11–18)	18 (12–19)	16 (14–18)	18 (18–18)	17 (14–18)	15 (15–18)	10.5 (5–17.5)	13.5 (9–17)	16 (13–19)
**Total score for FNPS** *Min. 10 Max. 50*	30 (24–35)	34 (23–39)	34 (30–35)	30.5 (26–35)	32 (28–36)	29 (26–31)	21.5 (14.5–34)	28 (22–33)	33 (27–38)
My confidence level in working with families is		3.5 (1–4)	3 (3–4)	2 (1–3)	3 (1–4)	3 (1–4)	2 (1–4)	3 (1–4)	4 (2–4)
My level of satisfaction with family care is		3.5 (1–4)	4 (3–4)	2.5 (1–4)	3 (1–4)	3 (2–4)	2 (1–4)	3 (1–4)	3 (2–4)
My knowledge level of family systems care is		3.5 (1–4)	4 (3–4)	2 (1–3)	4 (3–4)	2 (2–4)	3 (1–4)	3 (3–4)	3.5 (2–4)
My skill in working with the family system is		3.5 (3–4)	4 (3–4)	2 (1–3)	3 (2–4)	3 (2–4)	3 (1–4)	3 (3–4)	3.5 (3–4)
I feel comfortable in initiating family involvement in care planning		4 (1–4)	4 (3–4)	4 (4–4)	3 (1–4)	3 (1–4)	2 (1–4)	3 (1–4)	4 (2–4)
I plan care interventions in consultation with the patient and family		3 (1–4)	3 (3–4)	3.5 (3–4)	3 (2–4)	4 (2–4)	2 (1–4)	3 (1–4)	4 (3–4)
Families always approach me about their ill relatives		4 (3–4)	4 (2–4)	3.5 (3–4)	3 (1–4)	4 (3–4)	2 (1–4)	1.5 (1–4)	3 (1–4)
I promote patient/family participation, choice, and control in meeting health care needs		4 (3–4)	4 (3–4)	4 (4–4)	4 (3–4)	3 (3–4)	1 (1–3.5)	2.5 (1–4)	3.5 (1–4)
My involvement with families is mostly rewarding		3 (1–3)	4 (2–4)	3 (3–3)	4 (3–4)	3 (3–4)	2 (1–3.5)	3.5 (2–4)	3 (2–4)
I avoid interference of my own biases when collecting data about patients and families		4 (3–4)	3 (1–4)	4 (4–4)	4 (1–4)	3 (1–3)	2 (1–4)	3 (1–4)	4 (1–4)

* Family Nursing Practice Scale; low median (IQR) score 1–5; the lower the low score the higher the practice skill and reciprocity

### Internal reliability of the FINC-NA scale

The FINC-NA scale had an overall Cronbach’s alpha range from 0.63 to 0.79. The FAM-RNC subscale (resources in nursing care) had the lowest Cronbach’s alpha of 0.66. Further details are provided in Table 6.

**Table 6:** Cronbach’s alpha estimates for the FINC-NA and FNPS

Scale	Alpha
**FINC-NA scale**	
Family as a Resource in Nursing Care (Fam-RNC)	0.63
Family as a Conversational Partner (Fam-CP)	0.66
Family as a Burden (Fam-B)	0.6988
Family as Own Resource (Fam-OR)	0.79
**FNPS scale**	
Practice appraisal (PA)	0.73
Nurse-family reciprocity (NFR)	0.71

FINC-NA: Family Importance in Nursing Care- Nursing Attitude

FNPS: Family Nursing Practice Scale

### Healthcare providers perception of family care practices

**Fig. 1:**
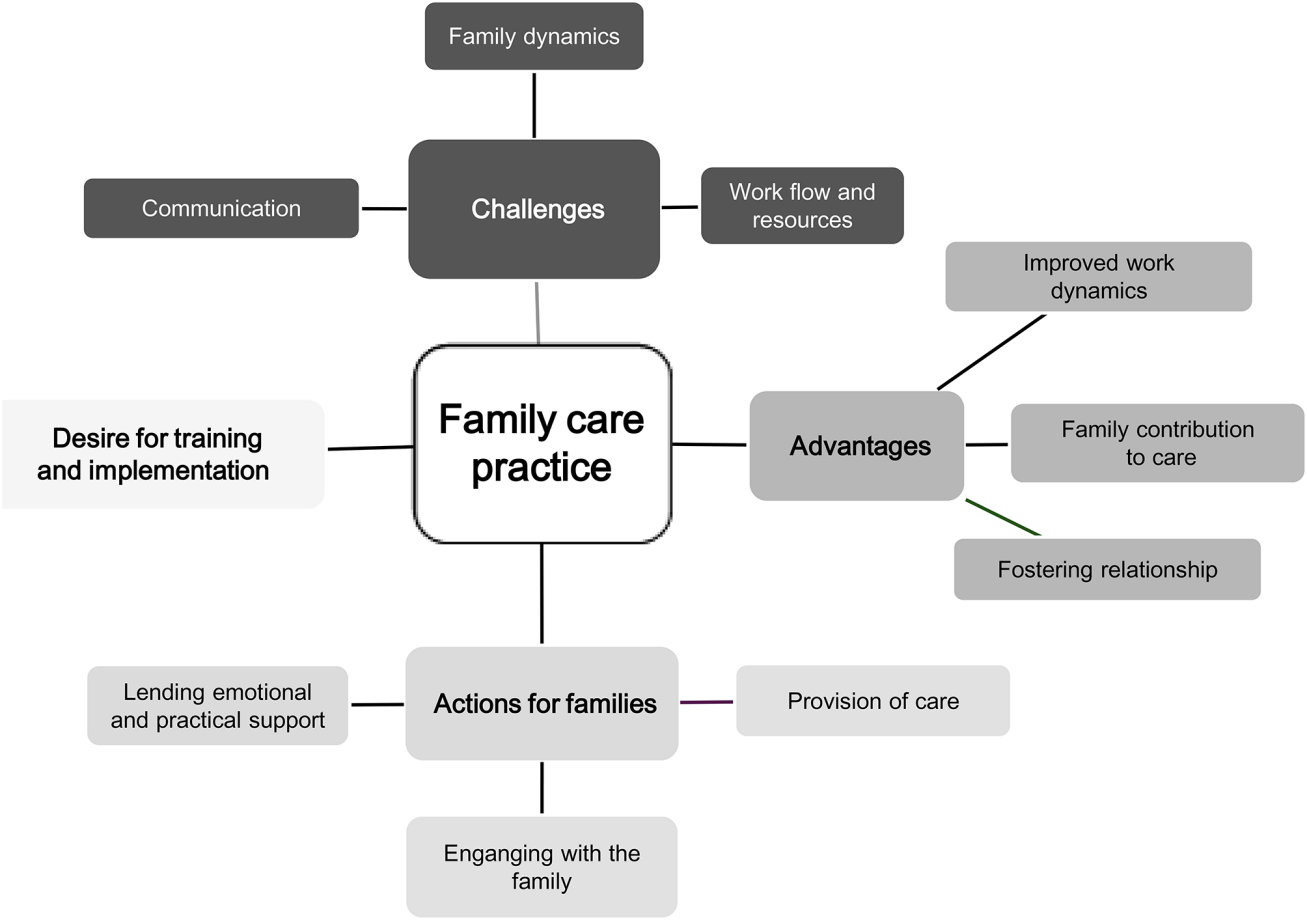
Healthcare providers’ perceptions on family care practices by four (4) categories and nine (9) sub-categories

Healthcare providers’ open-text responses regarding family care practices are synthesized into four main categories and nine subcategories (Fig. 1).

### Challenges for family care practice

Overall, 101 (74.3%) participants mentioned challenges with family involvement in assessment and care planning. Responses were grouped into three sub-categories

1. communication;
2. family dynamics;
3. workflow and resources.

#### Challenge 1: communication

The subcategory “communication” encompassed discrepancies in understanding regarding the care plan and included confidentiality issues. Language barriers, misunderstandings and difficulties with cooperation were mentioned. Healthcare providers recognized that patients and families often had opinions based on misconceptions about medical conditions. They also reported that family members were generally willing to share information but consistently preferred to do things “their way”. This was perceived as families not following instructions: *“They don’t do what they have been told to do”* [Medical assistant, CHPS] or *“They sometimes interfere with your work by overriding your decisions”.* [Nurse, health center]

Healthcare providers expressed confidentiality concerns in including families without prior consent as some medical conditions may lead to stigma or discrimination: *“The issue is confidentiality*,* especially when the patient has an underlying medical condition that could lead to discrimination”* [Medical doctor, labor ward]. Similar concerns resulted from inadequate space and challenging confidential discussions of sensitive information.

#### Challenge 2: family dynamics

Healthcare providers stated that the presence of family members sometimes makes patients (e.g., mothers) anxious when the family members themselves are worried. Healthcare providers also observed that when family members showed signs of worry, it prevented them from being an active part of the care process: *“Some family members become overly anxious*,* especially when their loved one is critically ill. This prevents them from participating in the care*” [Nurse, recovery ward].

Challenges and setbacks related to teenage pregnancy and male involvement were frequently reported.

#### Challenge 3: Workflow and resources

Several issues were raised concerning workflow, citing delays when involving families in the care process. One response highlighted the potential risk of decision-making delays caused by family involvement in the hospital process, and another feared that family members would distract healthcare providers.

Healthcare providers also pointed out that family members were difficult to involve due to their busy schedules: *“Sometimes due to work schedules*,* getting the family on board becomes a challenge*” [Midwife, CHPS].

Another typical challenge was family members providing herbal treatments to women in labor considered harmful by healthcare workers.

Resource constraints faced by families and the health system were frequently mentioned as a cause of family members’ absence and delays in payments of bills: “Sometimes they *(family members) will come in their numbers*,* but when it gets to the payment*,* they would all leave.”* [Nurse, health center].

Healthcare providers reported insufficient resources for equipment, particularly telecommunications. They were often even compelled to purchase some of these items with their personal funds.

### Advantages of family care practice

Overall, 120 (83.9%) healthcare providers perceived an advantage in family involvement in the assessment and care planning. Answers were grouped into three sub-categories: *(1) improved work dynamics*,* (2) family contribution to care*,* and (3) fostering relationship.*

#### Advantage 1: improved work dynamics

Involving families was mentioned to enhance communication and foster mutual understanding between healthcare providers and families. *“A client gets more understanding of her situation and*,* in turn*,* adheres to the (care) plan.”* [Midwife, postnatal unit].

Involving families in care was considered to improve information that optimizes care provision: *“Getting feedback from them enhances competent nursing care.”* [Nurse, health center].

Working with the family was also considered to support decision-making, and that ultimately translated into better care plan: *“Decisions made (together) with families are well implemented.*” [Nurse, health center].

Many respondents stated that involving families in the care had the potential to save time for healthcare providers. As an example, carrying out the physical examination of the newborn and the mother simultaneously was reported to facilitate work. Finally, collaborating with families was reported to influence their job satisfaction positively.

#### Advantage 2: family contribution to care

Healthcare providers reported that families can support their relatives with healthcare needs and also provide assistance with settling bills. During labor, family members were considered a source of comfort and support for the laboring woman. Families were felt to increase patients’ feeling of safety and trust in health care providers: *“It helps the patient to feel secure and have confidence in the caregiver.*” [Nurse, health centers].

Family involvement was considered to help achieve smooth and uninterrupted care and the overall health of newborns through better planning and care practices: *“It will improve the overall outcome for the neonates and minimize complications on discharge.”* [Nurse, NICU].

They also reported that family involvement promoted recovery: *“They (*family members*) support the client in the healing process.”* [Midwife, postnatal unit].

#### Advantage 3: fostering relationship

A few healthcare providers reported that involving families in the care may help build relationships between health professionals and families: *“It creates good interpersonal relationships between you (healthcare provider) and the family.”* [Midwife, labor ward].

A healthcare provider also mentioned that working with families may improve the relationship between the family and the child, as well as with other family members: *“It (the work with family) improves family-to-child relationships.”* [Nurse, CHPS].

### Actions for families

When healthcare providers were asked about actions taken in the past week to involve families in their current care practice, 121 (89.8%) responded with answers in the following three sub-categories:

*(1) provision of care*,* (2) engaging with the family and (3) lending emotional and practical support.*

#### Action 1: provision of care

Families were informed about the patient’s condition and counseled on the care plan: *“Called family members to inform them about the progress in the client’s condition and the necessary interventions to implement for further improvement.”* [Nurse, NICU].

Family members were used as a resource and instructed to administer medications and to provide support during labor, thereby enhancing overall well-being: *“I taught a relative how to provide diversional therapy to her sister in labor.”* [Midwife, labor ward].

Healthcare providers in the communities reported “*Educating families during home visits on care for newborns*,* puerperal women and pregnant women.”* [Midwife, health center] and also the general public: *“We held a durbar (public reception) in the community to involve nursing mothers and their husband and to educate them.”* [Nurse, CHPS].

#### Action 2: engaging with the family

Healthcare providers mostly involved families through phone calls, verbal communication and home visits. Families were encouraged to accompany the patient to the health facility. Also, specific counseling for male involvement was provided to support the decision-making process: *“Client’s relative (husband) was involved in planning mode of delivery for the pregnant woman.”* [Midwife, ANC].

Healthcare providers reported to ask for consent before involving the broader family.

#### Action 3: lending emotional and practical support

Healthcare providers reported to support through encouragement and by creating a protective environment to express fears and concerns. One healthcare provider mentioned: “*Always approach them (families) in a respectful manner.”,* [Nurse health center].

One way was to ask family members how they felt about their relative’s health. Suffering reportedly reduced through family involvement: “*A client who was admitted [at the hospital] was anxious and her relative was called to come and stay with her and the anxiety was gone.”* [Nurse, health center].

Practical help was also mentioned, for instance, for referrals between facilities.

### Desire for training and implementation

A key aspect highlighted by healthcare providers was the need for more training. Participants thought they had insufficient knowledge on family systems care in general and they asked for on-the-job training and making protocols available. One respondent thought that family systems care should be *“included in the yearly objectives”* of the institution.

## Discussion

Our study investigated healthcare providers’ views, attitudes and practice skills about the importance of families in the care of small and sick newborns along the care continuum in in Ghana. Key findings indicate overall good attitudes but limited practice skills while acknowledging challenges and benefits of involving families in the care process. Attitudes and skills varied though across units along the care continuum.

Attitudes of healthcare providers throughout the continuum of care, from the antenatal to postnatal care units were overall positive on family involvement. Numerous studies from high-income countries (Blöndal et al. 2014; Hagedoorn et al. 2021; Naef et al. 2020a; Naef, Kläusler-Troxler, Naef et al. 2020a, b), and rarely from LMICs (Imanipour and Kiwanuka 2020), have reported similar attitudes, leading to the conclusion that healthcare providers generally value the role of families in the care process. However, some qualitative studies from Ghana reported negative attitudes that resulted in conflicting relationships between healthcare providers and families, impacting the care of pregnant and birthing women and their newborns (Abukari and Schmollgruber 2024; Dugle et al. 2021; Nyande et al. 2024).

We observed that healthcare providers in the outpatient unit at the hospital had the highest positive attitude, together with healthcare providers from the neonatal intensive care unit. In contrast, primary care providers at the CHPS facility had the least positive attitudes towards family involvement. We hypothesize that these attitudes of healthcare providers at primary level result from a lack of training opportunities (Adusei et al. 2024). Our data confirms that these health providers had a lower educational level, which may also affect their ability to deliver effective FSC. These findings contrast with results from high-income countries where Hagedoorn et al. (2021) and Østergaard et al. (2020) found less family-friendly attitudes of nurses working in hospitals than in community care settings. However, not all found this difference (Cranley et al. 2022; Hoplock et al. 2019).

Answers to our open-ended questions confirmed that healthcare providers regarded families as a resource in the care process, and they encouraged their participation, aligning with findings from the structured FINC-NA instrument. Only staff from the labor ward and the CHPS perceived families as less of a resource. This finding may be attributed to staff dissatisfaction with prevailing conditions at labor wards and CHPS, thereby dwelling morale towards providing maternal and child care (Atinga et al. 2018; Dugle et al. 2021). During the intrapartum period, involvement was challenging due to limited space, intimacy, and trust issues, as reported by others from SSA (Ahmed et al. 2023), necessitating resolution if inclusive FSC is to be promoted.

Our participants perceived families as a moderate burden, similar to Malawi, where some healthcare providers considered families a hindrance (Mhango et al. 2020). Healthcare providers who consider family members burdensome tend to involve them less (Benzein et al. 2008b; Hsiao and Tsai 2015). While families are considered generally helpful, anxious family members may be perceived as demanding and prone to overstepping boundaries, thus explaining the opposite attitude.

Healthcare providers in our survey made use of available families’ own resources. Imanipour and Kiwanuka (2020) reported that most healthcare providers encouraged families to use their own coping resources. We found the lowest consideration for family as a resource among healthcare providers at CHPS and the postnatal unit, but not at health centers. In a multinational study, different practice areas also showed various results concerning family as a resource (Cranley et al. 2022). The low emphasis on family as a resource at CHPS facilities may be partly due to the anticipated fear of referrals in cases of serious illness that deters families from utilizing CHPS services. This leads health providers to engage less with families and underutilize strategies that tap into families’ inherent resources (Bassoumah et al. 2021; Schuler et al. 2023b). In the postnatal care unit, the lower use of family resources may result from the need to reinforce education on danger signs at home. Similar attitudes have been reported in Australia, Denmark (Dieperink et al. 2018) and South Africa (Emmamally and Brysiewicz 2019). Group counseling sessions, routinely employed in postnatal care units in Ghana (Owen et al. 2020), may thus stand somewhat in the way of individual emotional family support and promotion of coping mechanisms.

Healthcare providers acknowledged family members as dialogue partners, similar to Imanipour and Kiwanuka (2020) but contrary to Hagedoorn et al. (2021). In the open questions, healthcare providers reported that misunderstandings, language barriers and conflicting beliefs affected care. This is consistent with other studies supporting that effective communication and interaction between patients and healthcare providers is lacking globally, not only in SSA (Appiah et al. 2023; Kassa et al. 2020; Lythgoe et al. 2021). Language barriers have been reported in Ghana (Nyande et al. 2024; Ohene et al. 2022). Family members who do not understand instructions are often perceived as “difficult” (Nyande et al. 2022). Unclear information and ineffective communication during hospitalization may impact post-discharge care and outcomes (Abukari and Schmollgruber 2023).

A principal concern raised by healthcare providers was confidentiality, also pin-pointed by others, particularly during childbirth (Dzomeku et al. 2023) and pediatric care (Khumalo et al. 2020). Hence, patient consent must become an integral part of FSC.

As opposed to findings from an earlier study where healthcare providers had higher education and more exposure to FSC (Misto 2018), in our study, knowledge, confidence and skills in family systems care was generally low across the continuum of care. This contrasted with the high value attributed to family involvement in the care.

The lowest reported skill levels were among healthcare providers from the labor ward and the CHPS. Rural health facilities notoriously suffer from staffing and training shortages (Adusei et al. 2024). High workload and limited space in labor wards may also restrict opportunities to work closely with families (Dzomeku et al. 2022).

Grewal et al. (2024) showed a beneficial influence of the healthcare provider-family relationship, particularly for interventions in the neonatal period, while strengthening the relationship within the wider community after discharge may reduce the burden of morbidity and mortality in high-risk newborns. We believe this relationship may be built during antenatal care and continued during labor. We, therefore, suggest capacity building on FSC for labor ward and primary care staff to improve the healthcare provider-family relationship.

Healthcare providers’ views on the availability of the FSC in their wards were inconsistent. However, written policies appear to positively influence attitudes towards families and improve quality care (Barreto et al. 2022; Hagedoorn et al. 2021). Policymakers should develop policies to promote FSC in maternal and newborn care while health managements oversee the implementation (Imanipour and Kiwanuka 2020; Mirlashari et al. 2020).

We align with previous research in emphasizing the importance of education and research on the role of families in the care process as a critical component for improving care quality for women, newborns, and their families (Abukari and Schmollgruber 2024; Ahmed et al. 2023; Landry et al. 2023). Our findings underscore the urgent need for educational programs, in-service training, and decentralized training for staff in rural areas (Abukari and Schmollgruber 2024; Nyande et al. 2024).

Specifically for Ghana, our results support integrating FSC education into nursing, midwifery, and medicine curricula. This should prioritize the development of communication skills such as listening, respect, interpersonal skills and fostering mutual relationships (Shamali et al. 2022). Further research in Africa is essential to expand the evidence base and inform practice.

The strength of our study is the use of standardized questionnaires to investigate FSC and its cultural acceptance in a low-income context. Results may not be directly applicable as the FINC-NA and the FNPS scales were developed in and for high-income settings. But the scales are sufficiently adaptable to yield meaningful data when applied to new regions or settings. Complemented by open-text components, they offer rich insights along the entire care continuum of maternal and newborn health. Including all units along this continuum of care led to an unequal number of participants, as some have fewer staff. But, diversity and open answers enhanced robustness and depth, providing a comprehensive understanding of the topic from multiple perspectives.

One limitation is that our study was descriptive, and no analytical methods were applied. The generalizability might be limited due to the frequent turnover of healthcare providers in the study setting. As 80% of participants were female and male nurses reported having lower consideration for families as a resource (Benzein, Johansson, Arestedt, Berg, et al., 2008; Shamali et al. 2022). However, this reflects a real-life problem, as female/male ratios in the healthcare sector are unbalanced, particularly in maternal and newborn health (WHO 2024b).

## Conclusion

Healthcare providers acknowledged the crucial role families play in the continuum of care for small and sick newborns and expressed favorable attitudes towards family involvement. They identified several benefits, including stronger relationships between healthcare providers and families, improved treatment compliance and emotional support.

Healthcare providers’ abilities to effectively work with families were moderate, with several identified barriers: confidentiality issues and ineffective communication of care plans, thereby hindering relationships between healthcare providers and families and affecting intra-family dynamics.

To enhance family involvement throughout the care continuum, it appears essential to integrate FSC training into the curricula of nurses, midwives, and physicians, as our data confirms that healthcare workers are keen to use FSC but need education. In-service training may complement basic training and support the current workforce.

## Electronic supplementary material

Below is the link to the electronic supplementary material.

Supplementary Material 1

## Data Availability

No datasets were generated or analysed during the current study.

## Abbreviations

CHPS: Community based Health Planning and Services
CHN: Community Health Nurse
CoC: Continuum of Care
FINC-NA: Family Importance in Nursing Care- Nursing Care
FNPS: Family Nursing Practice Scale
IQR: Interquartile range
LMICs: Low- and Middle-Income Countries
NICU: Neonatal Intensive Care Unit
PHNU: Public Health and Nutrition Unit
SSA: Sub-Saharan Africa
