# Correction: Family involvement along the care continuum for small and sick newborns – attitudes and skills of healthcare providers in Ghana

**DOI:** 10.1186/s41043-026-01300-2

**Published:** 2026-04-18

**Authors:** Christina Schuler, Faith Agbozo, Emmanuel Bansah, Richard Owusu, George Edward Ntow, Barbara Preusse-Bleuler, Riccardo E. Pfister

**Affiliations:** 1 Institute of Global Health, Faculty of Medicine, University Geneva, Geneva, Switzerland; 2 Institute of Nursing, School of Health Sciences, ZHAW Zurich University of Applied Sciences, Winterthur, Switzerland; 3 Department of Family and Community Health, Fred N. Binka School of Public Health, University of Health and Allied Sciences, Ho, Ghana; 4 Volta Regional Hospital, Hohoe, Ghana; 5 Dodowa Health Research Centre, Dodowa, Ghana; 6 Neonatal and Pediatric Intensive Care Unit, University Hospitals of Geneva and Geneva University, Geneva, Switzerland

**Correction: Journal of Health, Population and Nutrition (2025) 44:277**

10.1186/s41043-025-01001-2

In the original publication of this article (Schuler et al. 2025), the authors identified the errors concerning the results of the Family Nursing Practice Scale (FNPS) in Abstract, Results, Discussion and Tables. The corrected texts and tables are presented with this correction.

In Abstract under results section, the first sentence should read, “Overall, 143 healthcare providers, comprising nurses, midwives and physicians, participated in the study. The median score of FINC-NA was 93 (83–99), while the FNPS was 22 (17–26)”.

In Results under sub-sections Healthcare providers’ appraisal of practice skills and reciprocity, the paragraph should read, “Table 5 details healthcare providers’ practice skills and the healthcare provider-family relationship. Lower scores indicate more effective practice or a stronger relationship. The overall score on the FNPS scale was 22 out of a maximum of 50. The highest practice skills were observed in the Public Health and Nutrition Unit (PHNU) (14.5), in the Antenatal Care Unit (ANC) (20.5) and in the health centers (20). The lowest practice skills were observed for the postnatal ward (25), and the Neonatal Intensive Care Unit (NICU) (NICU) (24), followed by the community-based Health and Planning Services (CHPS) (23) and the labor ward (22). In the subscale practice appraisal (PA), healthcare providers at the PHNU (7) and recovery ward (9.5) showed the highest critical appraisal of their own family care practice. The lowest skills were reported from the NICU (12) and the CHPS compounds (11.5). The PHNU had the highest interaction and reciprocity in healthcare provider-family relationship (NFR) (7.5), followed by the health centers (9). The lowest relationship competencies were reported from the postnatal ward (12), the recovery ward (12) and the NICU (12), showing moderate healthcare provider-family relationship”.

In the same section under heading Internal reliability of the FNPS scale, the first sentence should read, “The FNPS subscale Practice appraisal (PA) had a Cronbachs’s alpha of 0.84 and the Nurse Family Relationship (NFR) Score had a Cronbach’s alpha of 0.83”.

Under Discussion section, the paragraghs should read, “The lowest reported skill levels were among healthcare providers from the NICU, postnatal ward and the CHPS. Rural facilities and NICUs notoriously suffer from staffing and training shortages (Abukari & Schmollgruber, 2024; Reddy et al., 2022). High workload and limited space in NICUs and postnatal may also restrict opportunities to work closely with families (Abukari & Schmollgruber, 2024).

Grewal et al. (2024) showed a beneficial influence of the healthcare provider-family relationship, particularly for interventions in the neonatal period, while strengthening the relationship within the wider community after discharge may reduce the burden of morbidity and mortality in high-risk newborns. We believe this relationship may be built during antenatal care and continued during labor. We, therefore, suggest capacity building on FSC for postnatal and primary care staff to improve the healthcare provider-family relationship.

**Table 5:** Median scores for healthcare providers’ skills in working with families (FNPS) (*n* = 143)

Scale	Overall	ANC	Labor ward	Recovery ward	NICU	Postnatal ward	PHNU	Health center	CHPS
**Total score for FNPS** Min. 10 Max. 50	22 (17–26)	20.5 (17.5–22)	22 (18–25)	21.5 (19.8–23.2)	24 (20–28)	25 (21–27)	14.5 (11.8–20.5)	20 (16–23.8)	23 (20–28.8)
Health provider -family relationship (NFR) (Min.5 – Max.25)	10 (8–13)	10.5 (9.2–12)	11 (9–12)	12 (12–12)	12 (10–13)	12 (11–15)	7.5 (5–10)	9 (7–12)	10.5 (9–15)
Practice appraisal (PA) (Min 5 – Max 25)	10 (8–13)	10 (7.5–10.8)	10 (10–13)	9.5 (7.8–11.2)	12 (9–14)	10 (9–15)	7 (6.8–10.5)	10.5 (8–12.8)	11.5 (10–15)

Item		ANC	Labor ward	Recovery ward	NICU	Postnatal ward	PHNU	Health center	CHPS
My confidence level in working with families is		2 (1.2–2)	2 (1–3)	2 (1.5–2.5)	2 (1–3)	2 (2–3)	1.5 (1–2)	2 (1–2)	2 (2–3)
My level of satisfaction with family care is		2 (1–2)	2 (2–3)	1.5 (1.2–1.8)	2 (1–2)	2 (2–3)	1.5 (1–2)	2 (1–2.8)	2 (2–3)
My knowledge level of family system care is		2 (2–2)	2 (2–3)	2 (1.5–2.5)	2 (2–3)	2 (2–4)	2 (1–2.2)	2.5 (2–3)	2 (2–3)
My skill in working with the family system is		2 (2–2.8)	2 (2–3)	2 (1.5–2.5)	3 (2–4)	2 (2–3)	2 (1–2.2)	2 (2–3)	2 (2–3)
I feel comfortable in initiating family involvement in care planning		2 (1.2–2)	2 (2–2)	2 (2–2)	2 (1–2)	2 (2–3)	1.5 (1–2)	2 (1–2.8)	2 (2–2.8)
I plan care interventions in consultation with the patient and family		2 (1.2–2.8)	3 (2–3)	2.5 (2.2–2.8)	3 (2–4)	3 (2–4)	1.5 (1–2)	2 (1–3)	2 (2–3)
Families approach me about their ill relative		2 (1.2–2)	2 (2–2)	2.5 (2.2–2.8)	2 (1–2)	2 (2–3)	1.5 (1–2)	1.5 (1–2)	2 (1–2.8)
I promote patient/family participation, choice and control in meeting health care needs		2 (2–2)	2 (2–3)	2 (2–2)	2 (2–3)	3 (2–3)	1 (1–2)	2 (1–2)	2 (1.2–3)
My involvement with families is mostly rewarding		3 (1.2–3)	2 (2–2)	3 (3–3)	2 (2–3)	3 (2–3)	1.5 (1–2.2)	2 (2–3)	3 (2–3.8)
I avoid interference of my own biases when collecting, interpreting and communicating data about patients and families		2 (1.2–2)	2 (1–2)	2 (2–2)	2 (1–2)	2 (1–3)	1.5 (1–2)	2 (1–2)	2 (1.2–3)

*Family Nursing Practice Scale; low median (IQR) score 1–5; the lower the low score the higher the practice skill and reciprocity

ANC= Antenatal Care, NICU=Neonatal Intensive Care Unit, PHNU=Public Health and Nutrition Unit, CHPS=Community-Based Health Planning and Services

**Table 6:** Cronbach’s alpha estimates for the FINC-NA and FNPS

Scale	Cronbach’s alpha
Practice appraisal (PA)	0.84
Health provider -family relationship (NFR)	0.83

Reddy et al. (2022) was missing and should have been “Reddy B, Thomas S, Karachiwala B, Sadhu R, Iyer A, Sen G, Mehrtash H, Tun­çalp Ö. A scoping review of the impact of organisational factors on providers and related interventions in LMICs: Implications for respectful maternity care. PLOS Global Public Health. 2022;2(10):e0001134”.
