# Supplementary material for: Family involvement along the care continuum for small and sick newborns – attitudes and skills of healthcare providers in Ghana
Source: J Health Popul Nutr. 2025 Aug 4;44:277. doi: 10.1186/s41043-025-01001-2 (PMC12323203; doi:10.1186/s41043-025-01001-2)
Supplement: Supplementary file 1 — Supplementary Material 1 [file 41043_2025_1001_MOESM1_ESM.pdf]

STROBE Statement—checklist of items that should be included in reports of observational studies

	Item No.	Recommendation	Page No.	Relevant text from manuscript
**Title and abstract**	1	(*a*) Indicate the study's design with a commonly used term in the title or the abstract	1	See manuscript
		(*b*) Provide in the abstract an informative and balanced summary of what was done and what was found	1	See manuscript
**Introduction**				
Background/rationale	2	Explain the scientific background and rationale for the investigation being reported	3-5	
Objectives	3	State specific objectives, including any prespecified hypotheses	6-7	
**Methods**				
Study design	4	Present key elements of study design early in the paper	7	This descriptive cross-sectional study mixed-method study was based on an online survey. The survey was used, adhering to the Strengthening the Reporting of Observational Studies in Epidemiology (STROBE) guidelines for articles reporting cross-sectional studies (STROBE-Initiative, 2024).
Setting	5	Describe the setting, locations, and relevant dates, including periods of recruitment, exposure, follow-up, and data collection	7-8	
Participants	6	(*a*) *Cohort study*—Give the eligibility criteria, and the sources and methods of selection of participants. Describe methods of follow-up	NA	
		Case-control study—Give the eligibility criteria, and the sources and methods of case ascertainment and control selection. Give the rationale for the choice of cases and controls		
		Cross-sectional study—Give the eligibility criteria, and the sources and methods of selection of participants	8-9	
		(*b*) *Cohort study*—For matched studies, give matching criteria and number of exposed and unexposed	NA	
		Case-control study—For matched studies, give matching criteria and the number of controls per	NA (not a	

		case	matched study)	
Variables	7	Clearly define all outcomes, exposures, predictors, potential confounders, and effect modifiers. Give diagnostic criteria, if applicable	8-9	
Data sources/ measurement	8*	For each variable of interest, give sources of data and details of methods of assessment (measurement). Describe comparability of assessment methods if there is more than one group	8-9	
Bias	9	Describe any efforts to address potential sources of bias	NA	
Study size	10	Explain how the study size was arrived at	8	The population census approach was used to determine the sample size (Israeli, 1992). At the time of the study there were 208 healthcare providers working in the various units of the hospital and primary care structures. For this limited number of providers, we decided to invite all healthcare providers involved in maternal and newborn care.

Continued on next page

Quantitative variables	11	Explain how quantitative variables were handled in the analyses. If applicable, describe which groupings were chosen and why	NA	
Statistical methods	12	(*a*) Describe all statistical methods, including those used to control for confounding	10	
		(*b*) Describe any methods used to examine subgroups and interactions	NA	
		(*c*) Explain how missing data were addressed	No missing data	
		(*d*) *Cohort study*—If applicable, explain how loss to follow-up was addressed<br>*Case-control study*—If applicable, explain how matching of cases and controls was addressed<br>*Cross-sectional study*—If applicable, describe analytical methods taking account of sampling strategy	NA<br>NA<br>NA	
		(*e*) Describe any sensitivity analyses	NA	
**Results**				
Participants	13*	(a) Report numbers of individuals at each stage of study—eg numbers potentially eligible, examined for eligibility, confirmed eligible, included in the study, completing follow-up, and analysed	11	
		(b) Give reasons for non-participation at each stage	NA	
		(c) Consider use of a flow diagram	Not used	
Descriptive data	14*	(a) Give characteristics of study participants (eg demographic, clinical, social) and information on exposures and potential confounders	11 table 2	
		(b) Indicate number of participants with missing data for each variable of interest	NA	
		(c) *Cohort study*—Summarise follow-up time (eg, average and total amount)	NA	
Outcome data	15*	*Cohort study*—Report numbers of outcome events or summary measures over time	*NA*	
		Case-control study—Report numbers in each exposure category, or summary measures of exposure	*NA*	
		Cross-sectional study—Report numbers of outcome events or summary measures	*13-14*	
Main results	16	(*a*) Give unadjusted estimates and, if applicable, confounder-adjusted estimates and their precision (eg, 95% confidence interval). Make clear which confounders were adjusted for and why they were included	NA	
		(*b*) Report category boundaries when continuous variables were categorized	NA	Descriptive statistics
		(*c*) If relevant, consider translating estimates of relative risk into absolute risk for a meaningful time period	NA	

Continued on next page

Other analyses	17	Report other analyses done—eg analyses of subgroups and interactions, and sensitivity analyses	Open-ended questions 15-19	
**Discussion**				
Key results	18	Summarise key results with reference to study objectives	20	
Limitations	19	Discuss limitations of the study, taking into account sources of potential bias or imprecision. Discuss both direction and magnitude of any potential bias	23	
Interpretation	20	Give a cautious overall interpretation of results considering objectives, limitations, multiplicity of analyses, results from similar studies, and other relevant evidence	20-23	
Generalisability	21	Discuss the generalisability (external validity) of the study results	24	The generalizability might be limited due to frequent turnover of healthcare providers in the study setting.
**Other information**				
Funding	22	Give the source of funding and the role of the funders for the present study and, if applicable, for the original study on which the present article is based	25	Open access funding provided by Zurich University of Applied Sciences.

*Give information separately for cases and controls in case-control studies and, if applicable, for exposed and unexposed groups in cohort and cross-sectional studies.

Note: An Explanation and Elaboration article discusses each checklist item and gives methodological background and published examples of transparent reporting. The STROBE checklist is best used in conjunction with this article (freely available on the Web sites of PLoS Medicine at http://www.plosmedicine.org/, Annals of Internal Medicine at http://www.annals.org/, and Epidemiology at http://www.epidem.com/). Information on the STROBE Initiative is available at www.strobe-statement.org.
